# Arrayed functional genetic screenings in pluripotency reprogramming and differentiation

**DOI:** 10.1186/s13287-018-1124-6

**Published:** 2019-01-11

**Authors:** Rodrigo Alexandre Panepucci, Ildercílio Mota de Souza Lima

**Affiliations:** 1 Laboratory of Functional Biology (LFBio), Center for Cell-Based Therapy (CTC), Regional Blood Center of Ribeirão Preto, Rua Tenente Catão Roxo, 2501, Ribeirão Preto, SP CEP: 14051-140 Brazil; 2 Department of Genetics, Ribeirao Preto Medical School, University of São Paulo (FMRP-USP), Ribeirão Preto, SP Brazil

**Keywords:** Human embryonic stem cells, Pluripotent stem cells, Cell differentiation, Cellular reprogramming, RNA interference, microRNA, Fluorescence microscopy, Flow cytometry, High content screening, Arrayed functional screenings

## Abstract

Thoroughly understanding the molecular mechanisms responsible for the biological properties of pluripotent stem cells, as well as for the processes involved in reprograming, differentiation, and transition between Naïve and Primed pluripotent states, is of great interest in basic and applied research. Although pluripotent cells have been extensively characterized in terms of their transcriptome and miRNome, a comprehensive understanding of how these gene products specifically impact their biology, depends on gain- or loss-of-function experimental approaches capable to systematically interrogate their function. We review all studies carried up to date that used arrayed screening approaches to explore the function of these genetic elements on those biological contexts, using focused or genome-wide genetic libraries. We further discuss the limitations and advantages of approaches based on assays with population-level primary readouts, derived from single-parameter plate readers, or cell-level primary readouts, obtained using multiparametric flow cytometry or quantitative fluorescence microscopy (i.e., high-content screening). Finally, we discuss technical limitation and future perspectives, highlighting how the integration of screening data may lead to major advances in the field of stem cell research and therapy.

**Electronic supplementary material:**

The online version of this article (10.1186/s13287-018-1124-6) contains supplementary material, which is available to authorized users.

## Background

Pluripotency (the potential to differentiate in virtually all tissue cell types of the organism) and the ability to self-renew (to replicate maintaining the undifferentiated pluripotent state) are characteristic properties of embryonic stem cells (ESCs) [1].

At the beginning of embryogenesis, the zygote, surrounded by the zona pellucida, divides into two blastomers and, then, by successive divisions, originates the morula, which is reorganized to give rise to the blastocyst, composed (in its pre-implantation phase) of an outer layer called trophectoderm (TE) and an inner cell mass (ICM). At a later moment, the blastocyst hatches from the zona pellucida and TE cells differentiate into the trophoblast, which is responsible for the implantation of the embryo in the uterus; following implantation, ICM originates epiblast (EPI) and primitive endoderm (PrE or hypoblast) cells. In mice, TE cells contribute to the placenta, PrE to yolk sac, whereas EPI cells are considered pluripotent, as they originate cells from the three germ layers (ectoderm, endoderm, and mesoderm), as well as extraembryonic mesoderm [2].

Pluripotent cells can also be found in germ cell tumors, embryonal carcinomas (EC), or teratocarcinomas [3]. The identification and culturing of these cells led to the derivation of the first mouse embryonic stem cells (mESCs) lines, established from blastocyst ICM cells cultured on a layer of murine embryonic fibroblasts (MEFs), used as feeder cells, in the presence (or not) of culture medium conditioned by EC cells [4, 5]. The same was later done with human embryos, with the establishment of embryonic stem cells (hESCs) [6].

Several markers can be used to access the undifferentiated state or differentiation of ESCs [7] and the expression of many of these, including pluripotency-related transcription factors (e.g., OCT4/POU5F1 and NANOG), surface markers (such as SSEA3, SSEA4, TRA-1-60, and TRA-1-81), and a high alkaline phosphatase (AP) activity, are shared with EC cells, such as the human NTERA2 (NT2) line [8–10]. In addition, NT2 has a microRNA profile that resembles hESCs [11] and both can be induced to differentiate by all-trans retinoic acid (ATRA or RA), undergoing changes in microRNA and marker profile [12, 13].

The success of mESC derivation was due to the secretion of leukemia inhibitory factor (LIF) by MEFs [14], which activates JAK kinases and, consequently, the transcriptional factor (TF) STAT3, inhibiting differentiation and promoting survival and self-renewal [15, 16]. Mouse ESC derived from the ICM of pre-implantation embryos are considered to be in a “Naïve” state of pluripotency, defined by the ability of a cell to self-renew, maintaining the potential to differentiate without tendencies, contributing to all germ layers in the context of normal in vivo development [17].

On the other hand, EPI formed after implantation rapidly undergoes marked changes, with activation of the ERK pathway by FGF and repression of pluripotency markers (such as Rex1, Klf2, and Klf4), priming the EPI to respond to signals inducing the specification to different germ layers [17], a process likely controlled by the upstream activation of RAS [18]. As a consequence of the signaling acting on these cells in vivo, by the time of derivation, stem cells derived from the epiblast of blastulas already implanted in a pre-gastrulation phase (termed EpiSCs) depend on the presence of FGF2 (bFGF) and Activin [19, 20], while mESCs in culture depend on LIF (and also BMP4, in the absence of serum) [21]. In view of these differences, mESCs and EpiSCs are referred to as “Naïve” or “Primed,” respectively [22].

Human ESCs (hESCs) are derived from blastocysts obtained by in vitro fertilization, and based on multiple characteristics such as flat colony morphology, growth factor dependence (FGF2 and Activin) or the inactivation of one of the X chromosomes (in female-derived cells), they are considered more similar to mouse EpiSCs than mESCs [23].

Somatic cells can be reprogramed into induced pluripotent stem cells (iPSCs), by different combinations of pluripotency-related transcription factors (TFs), such as OCT4, SOX2, KLF4, and c-MYC (OSKM) [26] or OCT4, SOX2, NANOG, and LIN28 [27], which are able to regulate the transcription of a large number of factors (including themselves) involved in the control of pluripotency and differentiation [28, 29].

In addition to the broad transcriptional control mediated by TFs, microRNAs (miRNAs) have been recognized as major post-transcriptional regulators of gene expression. miRNAs are small (~ 22 nt) RNA molecules that bind to partially complementary target mRNAs, blocking their translation and/or leading to their degradation [30]. Currently, there are 1917 hairpin precursor miRs (pre-miRs) annotated in the human genome, according to the latest release of miRBase (release 22 March 2018), with 2879 corresponding mature miRNAs.

Despite the developments in functional genomics, with genome-wide methods capable of profiling the transcriptome, proteome, and miRNome of different pluripotent stem cells under distinct situations, a true understanding of the molecular mechanisms responsible for the biological properties of these cells, as well as for the processes involved in reprograming, differentiation, and transition between Naïve and Primed states, depends on functional assays capable of systematically interrogating the roles of specific genes or their products (mRNAs, proteins, or miRS).

In this review, we describe distinct sets of molecular and genetic tools, and arrayed screening approaches, which allow gain- or loss-of-function studies to be carried in order to systematically evaluate the function of gene transcripts and miRs in such biological contexts, ranging from focused to genome-wide screens.

## Molecular biology tools for gain- or loss-of-function studies

In general, genes that host miRs are transcribed by RNA polymerase II, giving rise to primary miRs (pri-miRs). These pri-miRs assume a secondary structure resulting from the formation of self-complementary hairpins, being further processed in the nucleus by proteins including the DROSHA/DGCR8 complex, giving rise to pre-miRs that consist of a hairpin [31]. Alternatively, the pre-miRs can originate from introns of coding mRNAs, called miRtrons [32]. These pre-miRs are exported by the Protein Exportin 5 into the cytoplasm, where the hairpin loop is processed by Dicer, generating the mature miR duplex composed of two strands, one derived from the 5′ end and other from the 3′ end, respectively originating the -5p and -3p suffixes in miR names [33].

This duplex is then loaded into the RNA-induced silencing complex (RISC), containing the Argonaute protein, and while one strand is removed during the assembly process and degraded thereafter (referred as passenger strand or star “*” strand), the other one guides the complex to target mRNA transcripts (thus named guide strand), by the complementarity between the microRNA and its target site(s), predominantly, destabilizing and reducing their levels and/or inhibiting their translation [30, 34]. Multiple mechanisms mediate miR effects, including deadenylation, decapping and 5′–3′ decay, direct translation repression, and if the complementarity between the miR and its target site is complete, site cleavage [35].

Similar to the mechanism of action described for microRNAs, other double-stranded molecules (naturally generated endogenously, by different mechanisms, or artificially introduced in the cell) can bind to and down modulate a specific transcript by a mechanism called RNA interference (RNAi). Among other methods, RNAi can be attained by short hairpin RNA molecules (shRNAs), synthetic or transcribed by expression vectors (usually viral), by small synthetic double-stranded RNA molecules (small interfering RNAs, siRNAs) or by endoribonuclease-prepared siRNAs (esiRNAs). Similarly, microRNAs can also be synthesized or cloned in vectors for expression.

While first-generation vector-delivered shRNA libraries were modeled after pre-miRs [36], second-generation libraries were modeled after pri-miRs (pri-miR-30 specifically) [37]. These later libraries (also referred as Hannon-Elledge libraries) were shown to enter the RNAi pathway through a more natural route, leading to a more effective knockdown. In addition, the library cassettes could be easily shuttled into vectors containing different promoters or for different types of viral delivery, thus allowing these shRNAs to be used in a transient, constitutive, or inducible fashion and, also, increasing transduction efficiency for different cell types [38].

Once introduced in the cell, by lipotransfection, electroporation, or viral transduction, these molecules can be processed by the microRNA processing machinery, including Drosha/DGCR8 and/or Dicer complexes (depending on the design of the vector driving the expression of the shRNA or pri/pre-miR) and/or directly associate with RISC (in the case of e/siRNAs or synthetic miR mimics), mediating cleavage or inhibition, depending on its complementarity (as described). For a complete review regarding design and specificities of different RNAi strategies, see references [39, 40] and the section discussing design-related issues on gain- or loss-of-function tools. Some companies have entire genome-wide libraries readily available in 96- or 384-well plates (see Tables 1 and 2 and Additional file 1: Table S1), see reference for a historical perspective on the development of RNAi libraries [40].

**Table 1:** Functional RNAi arrayed screenings

Targeted genes/RNAi method	Cell model	Screening method	Readouts	No of hits (examples); additional findings. #	Refs.
326 cDNAs of M13 mESC subtracted with MEFs/LT or EP RNAi plasmids	Mouse ESCs	qPCR (DNA Engine Opticon 2 system, Bio-Rad)	qPCR of Oct4	8 (Zfp42/Rex-1, Pcna, Tle4, Dppa5, Wdr77, Ash2l, Uble1b).	[60]
70 mouse genes downregulated upon RA differentiation/LV shRNA	Mouse ESC line CCE	Manual FC	Self-renewal competition assay	10 (Esrrb, Tbx3, Tcl1, Nanog, Oct4 and Sox2).	[61]
1008 mouse genes encoding chromatin proteins/LT esiRNAs	Mouse ESC lines	Manual TLM	Defects in viability and alterations in cell or colony morphology	68, 20 validated (Tip60-p400 complex: Ruvbl1c, Ruvbl2c, Dmap1c, Ep400, Htatipc/Tip60, Trrapc. Yeats4).	[62]
197 chromatin regulators/LV shRNA (6 per gene)	Mouse ESCs	HCS (ArrayScan HCS Reader, Cellomics).	Average Oct4 staining intensity (Hoechst cells)	10 (SetDB1/ESET, Ube2i/Ubc9, Ehmt1, Suv39h2, Cbx7, Cbx8/Pc3, Ezh2, Hdac3, Sin3a, Sap18). *3 (Smarcd1, Arid1a, Smarcb1)*	[64]
25,057 mouse genome-scale library/LT esiRNAs	Mouse Oct4-Gip ESCs (Oct4-GFP)	Automated FC (FACSCalibur, BD)	% Oct4-GFP+ cells	282, 21 validated (Ctr9, Rtf1, Nfya, Ptbp1, Wdr61, Cpsf3, Fip1l1, Iws1, Thoc2, Rnf2, Cxxc1, Cnot1, Ncl, Apc, Ube2m, Shfdg1).	[74]
16,683 mouse siGenome library (Dharmacon)/LT siRNAs pools	Mouse Oct4-Gip ESCs (Oct4-GFP)	Automated FC (LSRII, BD)	% Oct4-GFP+ cells	148, 8 validated (Cnot3, Eny2, Fip1l1, Mga, Pcgf6, Pcid2, Smc1a, Trim28).	[75]
312 mouse genes enriched for gene targets involved in chromatin regulation, early developmental signal transduction, and transcription (Qiagen)/LT shRNA	Mouse Nanog-GFP NG4 ESCs cells	Automated FC (FACS sort or LSR II, BD)	% Nanog-GFP+ cells	19 (Smad2, Smad4, Ctnnb1, Nfkbia, Nfkbib, Jak1, Gsk3b, Stat3, Esrrb, Oct4, Sall4, Sox2, Rif1). *21 (Fgfr2, Fgfr4, Erk1, Axin2, Hira, Ptma, Raf1, RhoA, Smad7, Socs3, Tcf3, Smarca4, Smarcb1, Smarcc1, Smarce1)*.	[65]
**&** 30,892 cDNAs expression libraries, mouse (7249) and human (3820 from MGC and 19,823 from OriGene Technologies)/LT plasmid	Mouse P19 EC cells (Nanog-luciferase)	Automated L (CLIPR, Molecular Devices)	Nanog-luciferase Activity/Well	159, 90 validated (Timp2, Hig2, Mki67ip). *70, 14 validated (PU.1/Spi1, Prkaca, Jun)*.	[84]
21,121 human whole genome (Dharmacon)/LT siRNAs pools	Human H1 ESCs (Oct4-GFP)	Automated CFM (IXU Ultra, Research Instruments)	% Oct4-GFP+ cells and nuclei (Hoechst) count	566 (PRMD14, NFRKB, Med12/13/14/17/17/24, CDC42, RRAS, COL4A4, RHOA, CLASP1, AHCTF1, NUP107, TUBB4, PSMD2, ODF2, CENPA, DCTN2, APITD1, CLASP1, CENPQ, RAD9B, RAD17); reduced reprogramming (PRDM14, NFRKB).	[82]
10,000 mouse genes / LV shRNA	Mouse ESCs	HCS (ArrayScan, Cellomics)	Average Oct4 pixel intensity of cells (nuclei/Hoechst) in the well	43 (12 Med subunits, Smc1a, Smc3, Stag2, Nipbl).	[76]
4010 mouse genes, including all signaling genes, transcription factors, and chromatin regulators (Dharmacon)/LT siRNAs pools	**$** MEFs Reprograming (Dox-Inducible OKMS cassette)	HCS (InCell Analyzer 1000, GE)	Total iPSC Colony Area (overlap of AP/DAPI staining)	Not specified by authors (Cdh1, Par3 and Crb3, Smad1/4, BMPRII, ALK3).	[87]
571 kinases and 253 phosphatases/LT siRNA (Ambion Silencer libraries, Applied Biosystems)	Mouse ESCs CGR8 (with a Wnt/β-catenin signaling luciferase)	EnVision 2104 Multilabel Plate Reader (PerkinElmer).	Luciferase intensity	55 (AKT1, AURKA, CKB, TEC, MTMR6) decreased Wnt/β-catenin signaling. 14 (Dusp9, Dusp23, Dusp28) increased Wnt/β-catenin signaling.	[66]
319 human genes enriched in human NTera2D1 EC and H9 ESC lines (Dharmacon)/LT siRNA	Human NTera2D1 EC cells	Automated F	Relative cell number (Hoechst Intensity/Well)	23 (ZIC3, NANOG, SOX2, HMGA1, POU5F1, NR5A2, RBBP9, BIRC5, CDC2).	[81]
929 mouse kinases and phosphatases/4801 RV shRNAs	Mouse D3 ESCs	Automated SP	Relative cell number (Alamar Blue Intensity/Well), AP/Alamar Blue Ratio	27 (Nme6 and Nme7).	[67]
16,873 mouse siGENOME library (Dharmacon) / LT siRNAs pools	**@** Mouse Rex1GFPd2 ESCs (rex1-GFP, a Naïve Marker)	Automated FC (LSR II, BD)	Ratio of high (Naïve) to low (Primed) rex1-GFP expressing cells	130 (esrrb, stat3, ctr9, smc1a). *792, 316 validated (tcf7l1/tcf3, jarid2, dpy30, jun, mbd3, Gli3).*	[77]
640 known and predicted genes from the ubiquitin-proteasome system / LT siRNA pools	Mouse Nanog-GFP NG4 mESCs cells cultured in +LIF media or -LIF + RA media	Automated FC (FACS sort or LSR II, BD)	% Nanog-GFP+ cells	+LIF 20 (Psmd14 and Usp9x, Rbx1, Rfwd2, Rnf12, Ubr5, and Ddb1, Trim28, Phf5a); reduced reprogramming (Psmd14). *-LIF + RA 17 Nine (Fbxw7, Rnf152, Rnf31, Rnf8, Socs3, Topors, Rnf36, Tnfrsf25, Ubl5); increased reprogramming (Fbxw7).*	[72]
104 mouse ESC-associated phosphoregulators/LV shRNA	Mouse ESC line CCE	Automated FC (LSR II, BD)	Self-renewal competition assay	15 (Acvr2a, Aurka, Aurkb, Bub1b, Chek1, Dyrk3, Mapk4, Mapk13, Ppp4c, Ppm1g, Ppp2r1b); reduced reprogramming (Aurka)	[68, 69]
8296 and 1640 mouse genes, targeting, respectively, druggable genome and transcription factors (QIAGEN) / LT siRNA	**@** Mouse Oct4-Gip ESC (Oct4-GFP) in 2i media (Naïve)	Automated F (BioTek Flx800)	Relative number of puromycin-resistant (Oct4+) cells (Alamar Blue Intensity/Well)	*46, 28 validated (Raf1, Apc, Hgs, KRas, Mapksp1, Tcf7l1/Tcf3, Ctbp2, Tsc1/2, Flcn)*	[86]
16,872 mouse siGENOME library (Dharmacon)/LT siRNAs pools	Mouse Nanog-GFP NG4 ESCs cells	HCS confocal (ImageXpress Ultra, Molecular Devices)	% Nanog-GFP+ cells and nuclei (Hoechst) count	429, 9 validated (Ap2m1, Piwil2, Fbxw2, Helz2, Snai1, Brd4, Chd4, Htatip2, Prmt1); reduced reprogramming (Snail1). *299, 4 Validated (Brd8, Snai2, Hirip3, Ecd); increased reprogramming (Snai2).*	[78]
734 mouse kinase genes/3686 LV shRNA	**$** MEFs Oct4-GFP Reprograming (OSKM retroviruses)	Automated CFM (LSM710, Zeiss)	GFP+ iPSC Colony Count	59 (TESK1, LIMK2, DGKε, PLK2, BMP2K, BMPR2, MAPK1).	[89]
20 histone demethylases (HDMs)/LV 5 shRNA per gene	Mouse ESCs	Manual TLM	“ESC growth phenotype” and “colony morphology”	5 (Jmjd2b/Kdm4b, Jmjd2c/Kdm4c); reduced reprogramming.	[63]
1066 ubiquitin-proteasome system—UPS/LT 4 siRNA for each gene	* Mouse ESCs DTC26 (aMHC-GFP, cardiac-specific promoter of a-myosin heavy chain)	HCS (ImageXpress Micro, Molecular Devices)	Total GFP Area	10 (Fbx116, Wdr31, Rcbtb2, Stam2, Dpf3, Fbxl20, Cul5, Kcns3, Sumo2, Amfr).	[73]
652 epigenetic regulators/siRNAs	**$** MEFs Reprograming (Dox-induced OSKM)	HCS (InCell Analyzer 1000, GE)	Total iPSC Colony Area (overlap of AP/DAPI staining)	76 (Trrap, Ccdc101, Taf12, Gcn5; Ezh2, Suz12, Wdr5, Sirt6, Prmt5).	[88]
12,348 mouse genes/LT esiRNAs	**@** Mouse Oct4-GFP EpiSCs line OE7	Automated FC (FACSCalibur, BD)	% Oct4-GFP+ Naïve mESC	467, 53 validated (Apc, Ep300, Tox4, Med12/14, Ctr9, Rtf1, Wdr61/82, Cpsf3, Fip1l1, Cnot1/2/3, Tcf3, Cxxc1, Rnf2, Mll2). *149, 54 validated Brd4, Ctnnb1, Ep400, Smc1a, Rad21, Max, Tip60, Trrap).*	[80]
247 RNA-binding proteins—RBPs expressed in mESCs/LT 4 siRNAs for each target; Block-It, Invitrogen)	Mouse R1 ESCs	qRT-PCR (StepOnePlus System, Applied Biosystems)	qPCR for pluripotency (Nanog) and differentiation markers (Fgf5 / embryonic, Cdx2 / extraembryonic)	16 (Ddx47, Ddx52, Ddx56, Krr1, Nifk, Nol6, Pdcd11, Rbm28,Rrp7a; Cwc15, Hnrnpk, Hnrnpu, Ptbp1, Rbm42, Srsf7/9G8, Tardbp); reduced reprogramming (Krr1, Ddx47).	[70]
4558 human genes/siRNAs (Ambion / Dharmacon)	Human NANOG-GFP H1 ESCs cultured under 5 differentiation media: -bFGF-TGF-b, TGF-b inhibition, MEK inhibition, PI3K inhibition and Retinoic Acid	HCS confocal (ImageXpress Ultra, Molecular Devices)	% Nanog-GFP+ cells and nuclei (Hoechst) count	From 39 to 182 hits in 5 screening conditions (AURKA, PLK1, CDC25A, CDK1, CCNB1, CCNB2).	[83]
21,121 human genes targeted by siRNAs (whole-genome Dharmacon SMARTpooled siRNA library)/	**$** Human BJ fibroblast Reprograming (LV OSKM)	HCS confocal (ImageXpress Ultra, Molecular Devices)	TRA-1-60+ area per number of cells.	557, 39 validated (PPRC1, ENY2, MGA, PWP1, RAD21). *599, 37 validated (SMAD3, ZMYM2, SFRS11, SAE1, ESET).*	[93]
356 putative RNA-binding proteins—RBPs (SMARTpool siGENOME siRNAs)/LT	**@** Mouse ESCs with mCherry knocked into mir-290 ∼ 295 locus (Naïve marker) and eGFP into mir-302 ∼ 367 locus (EpiSCs marker)	HCS (INCell Analyzer 2000, GE)	Area of eGFP and mCherry normalized by Hoechst area.	*34 (adar, apex1, eif2a, elavl1, fus)* miR-302 reduction / inhibition of the ESC-to-EpiLC transition. 1 (NF45/Ilf2) miR-302 increase.	[71]

*Notes*: small interfering RNAs (siRNAs), short hairpin RNAs (shRNAs), endoribonuclease-prepared siRNAs (esiRNAs), O (Oct4), K (Klf4), M (c-Myc), S (sox2), lentiviral transduction (LV), retroviral transduction (RV), lipid transfection (LT), electroporation (EP), embryonal carcinoma (EC), embryonic stem cell (ESC), induced pluripotent stem cells (iPSCs), mouse embryonic fibroblasts (MEFs), epiblast stem cells (EpiSCs), flow cytometry (FC), spectrophotometry (SP), fluorometry (F), luminometry (L), transmitted light microscopy (TLM), fluorescence microscopy (FM), confocal fluorescence microscopy (CFM), high-content screening (HCS), alkaline phosphatase (AP). **#** Knockdown (KD) effect was usually associated with loss of pluripotency features, Naïve-Primed transition, differentiation induction or reduced reprograming (“Pluripotency/Reprograming Effectors” or “Differentiation Inhibitors”); however, some studies also identified genes whose KD promoted pluripotency or reduced differentiation (“Pluripotency/Reprograming Repressors” or “Differentiation Inducers,” italicized in the table). Knockdown effect on reprograming efficiency (when evaluated) is mentioned with the specific gene tested in parenthesis. & Gain of function screen. $ Reprograming. * Differentiation. @ Naïve-Primed Transition

**Table 2:** Functional microRNA arrayed screenings

Library/method	Cell model	Screening method	Readouts	No of hits (examples) #	Refs.
266 mouse miRNAs (Dharmacon)/LT SM	Mouse ESC DGCR8 knockout	Manual SP	Relative cell number (MTT assay)	14 (miR-19a, miR-20a/b, miR-33, miR-93, miR-106a, miR-223, miR-291a/b-3p, miR-294, miR-295, miR-302b/c/d)	[96]
379 mouse miRNAs (Ambion)/LT SM	**$** MEFs from OG2 mice (Oct4-GFP) Reprograming (OKS cassette lentiviral)	Semi-Automated FM	Oct4-GFP+ iPSC Colony counts	27, 14 validated (miR-130a/b, −148a, − 152, − 190, − 301, −301b, −669b, − 721 and miR-290 and miR-302 cluster members); siRNA KD of 130/301/721 family target *Meox2* increased reprograming.	[90]
52 candidates ESC-specific miRNAs/EP Amaxa, PB-CAG-miRNA piggybac vector	**$** MEFs (Oct4-GFP-Puro) Reprograming (PB-CAG-OCKS transposon)	Manual FC (Cytomics FC500 series, Beckman Coulter)	Resistance to Puromycin and % of GFP+ cells.	4 (miR-302 cluster, miR-25, miR-290, miR-298); miR-25 targets *Wwp2 and Fbxw7.*	[101]
875 human miRs (Ambion)/LT SM	* Mouse ESCs Myh6-eGFP (cardiac-specific)	HCS (InCell Analyzer 1000, GE)	Integrated fluorescence intensity of Myh6-eGFP	*19 (let-7a, let-7b, let-7c, let-7d, let-7e, let-7f, let-7 g, let-7i, miR-98, mus-miR-202, miR-18a, miR-18b*); KD of *let-7 and miR-18* targets (Acvr1b and Smad2, respectively) promoted mesoderm differentiation.	[103, 104]
570 Mouse miRNAs (Dharmacon)/LT SM	**$** MEFs from OG2 mice (Oct4-GFP) Reprograming (OSK cassette retrovirus)	HCS (InCell Analyzer 2000, GE)	Oct4-GFP+ iPSC Colony counts	16 (miRs − 294, −302a/b/d, −467d,-181b/d,-19a*, −34c*, −467d, − 294, − 677, − 451, −30d, − 590-3p, − 144, − 324-3p, − 455-5p). siRNA KD of miR-294 targets (*Cdkn1a, Zfp148, Hivep2, Ddhd1, Dpysl2, Pten, Cfl2, 9530068E07Rik*) or miR-181 targets (*Bptf, Lin7c, Cpsf6, Nr2c2, Bclaf1, Nol8, Igf2bp2, Marcks*) increased reprograming.	[102]
40 mouse miRNAs (Dharmacon)/LT SM	Mouse ESC DGCR8 knockout	Manual (varied)	AP colony staining, morphology, cell cycle, gene expression pattern and Oct4 staining	*14 (miR-218-5p, 200c-3p, 129-5p, 135b-5p, 24-3p, 9-5p, 32-5p and 27a-3p); miR-27a/24* targets Oct4, Foxo1, gp130, Smads; increased reprogramming (anti-miRs − 27a and − 24).	[97]
21 miR highly expressed or significantly upregulated during ESCs to EpiLC transition/LT SM	**@** Dgcr8−/− Naïve mESCs to EpiSCs transition	Manual qRT-PCR	Naïve markers Rex1 and Klf2 and epiblast marker Fgf5	*7 (miR-291a-3p, miR-294-3p, miR-295-3p, miR-302a/b/c/d-3p)*; siRNA KD of *miR-290/302 family* target Akt1 promoted Naïve to Primed transition..	[98]
379 mouse miRNAs (Ambion)/LT SM	**$** MEFs from OG2 mice (Oct4-GFP) Reprograming (OKS cassette lentiviral)	Semi-Automated FM	Oct4-GFP+ iPSC Colony counts	*14 (miRNA-212/132)*; siRNA KD of *miRNA-212/132* targets p300 and Jarid1a hampers reprogramming.	[92]
31 human miRNAs differentially expressed upon differentiation of pluripotent cells (Ambion)/LT SM	Human NTERA2 EC and Human H1 ESCs	Manual HCS (ImageXpress Micro, Molecular Devices)	Multiparametric Phenotypic Profile (Hoechst/CellMask, anti-Oct4 and – CycB1)	*Lineage dependent (miR-22-3p, miR-27a-3p, miR-29a/b, miR-30a-5p)* Lineage dependent (miR-106a-5p, miR-302a/b/c/d-3p, miR-372-3p and miR-373-3p; miR-92a-3p, miR-363-3p); miR-363 targets *NOTCH1 and PSEN1*	(submitted, 2018)

*Notes*: lipid transfection (LT), electroporation (EP), synthetic mimics (SM), flow cytometry (FC), spectrophotometry (SP), fluorometry (F), luminometry (L), transmitted light microscopy (TLM), fluorescence microscopy (FM), confocal fluorescence microscopy (CFM), high-content screening (HCS), alkaline phosphatase (AP). **#** Gain of function effect of introduced miRs could promote pluripotency/reprograming or inhibit Naïve-Primed transition or differentiation (“Pluripotency/Reprograming Effectors” or “Differentiation Inhibitors”); alternatively, miRs could be associated with reduced pluripotency/reprograming, Naïve-Primed transition or differentiation induction (“Pluripotency/Reprograming Repressors” or “Differentiation Inducers,” italicized in the table). Effect on reprograming efficiency (when evaluated) is mentioned with the specific miRNA (or anti-miR/inhibitor) tested in parenthesis. $ Reprograming. * Differentiation. @ Naïve-Primed Transition

In the case of microRNAs, several studies allowed the identification of the sequence elements determining the specificity of miR:mRNA interactions, stablishing algorithm scores that can predict target sites in mRNA transcripts and, additionally, determine a score that correlates linearly with the amplitude of the expected regulation at the mRNA level [34, 41, 42]. Based on these prediction tools, once a microRNA of interest is found, its predicted targets can be identified and a secondary focused siRNA screen against them can be carried to verify if the phenotype induced by the microRNA is, at least in part, mediated by one or more specific targets.

In the case of miRs, their forced expression (or introduction of mimics) leads to a gain-of-function, however, when a given miR is expressed endogenously by the cell, its function can be inhibited with the use of different strategies. One strategy uses inhibitory molecules, called anti-miR, that correspond to single-stranded synthetic RNA molecules designed and/or chemically modified to bind with high specificity and affinity to the corresponding endogenous mature miR. As a consequence endogenous miRs are sequestered, resulting in increased translation of their targets and/or increased levels of target transcripts [43–45].

## Functional arrayed screenings

Arrayed functional screenings comprise the use of spatially separated reagents arranged in multiwell microplates (e.g., a single molecule from a genetic library per well), allowing the effect of each reagent to be independently evaluated on cells (in a particular biological context). In order to reduce costs and to increase throughput, assays are usually miniaturized to be carried in 96-, 384-, or 1536-well plates, what usually demands special facilities to automate liquid and plate handling, to allow a timely and reproducible execution of the screen [46, 47].

A key criterion for the success of cell-based assays is the use of cell models compatible with the technical restrictions associated with the small volumes of the culture microplates, which limits nutrient availability, preventing prolonged cultures. As a result, assays must be preferentially carried out in short time frames. While mouse ESCs are relatively easy to culture and to manipulate, in the context of automated arrayed screenings, human ESC lines are technically difficult to be handled and maintained in culture. On the other hand, pluripotent cell lines derived from embryonal carcinomas (EC) can be easily grown [48] and are considered classic experimental models for in vitro studies of mechanisms related to self-renewal and differentiation [49].

In order to carry cellular assays in a reproductive way (a crucial condition for the reliable quantification of the parameters evaluated), cells must be submitted to all treatment conditions as simultaneous as possible. In the case of libraries of synthetic molecules (e.g., siRNAs or miR mimics), usually cells are reverse transfected. For this, each molecule of the library is pre-dispensed in a given well of an assay culture plate (using pipetting robots or acoustic delivery systems) and, then, a liquid dispenser (usually a peristaltic pump) is used, first to dispense the transfection reagents into wells (allowing it to mix with the RNA molecule) and, next, to dispense cells and culture media on these same wells.

Cells are then cultured for a certain period of time and submitted to a plate reader capable of quantifying a given parameter associated with the biological function being evaluated. Several approaches can be used to obtain functional readouts of an arrayed screening, but, in general, they can be divided in two main categories: those whose methodology restricts the quantitation of parameters (usually one or few) to a single value representing the whole population (which we denominate “population-level primary readouts”), and those that allow the quantitation of multiple parameters at a single-cell level (which we denominate “single-cell-level primary readouts”).

### Population-level primary readouts

Those in the first category are also referred as biochemical readouts, as they may rely on the use of reagents or enzyme substrates whose optical properties are modified upon processing by a given enzyme or upon reaction with a cell substance. For instance, tetrazolium salts (XTT and MTT) are widely used to evaluate viability and proliferation spectrophotometrically, based on their conversion to an insoluble purple or water-soluble orange formazan product, respectively, by mitochondrial dehydrogenase [50]. Resazurin (Alamar Blue), a blue dye that becomes pink and fluorescent upon reduction to resorufin, can also be used to quantify cell viability using a plate spectrometer or, more sensitively, a fluorimeter [51, 52]. Assays based on cell lines harboring reporter genes, such as luciferase and GFP, can also be used to derive population-level measurements (read by luminometers or fluorimeters, respectively), without the need for complex staining procedures [53].

### Single-cell-level primary readouts

Although the simpler characteristics of assays based on population-level readouts allow a less cumbersome execution of high-throughput screening (HTS) campaigns, given the heterogeneous nature of pluripotent stem cell cultures, experimental approaches based on single-cell-level primary readouts are particularly suited to explore this variability, by allowing the quantitation of multiple parameters at the single-cell level, as well as at the population level [53]. Two main techniques can give this type of multiparametric readouts.

Flow cytometry allow the quantitation of many markers at a single-cell level (depending on the combinations of fluorophores and excitation/emission channels); however, it can only provide a couple of morphological measurements, associated with the size (Forward SCatter, FSC) and internal complexity/granularity of the cell (Side SCatter, SSC). Moreover, cells must be acquired in suspension, what removes any spatial information that may exist in 3D or 2D adherent cultures [54].

Recently, the technology collectively referred as high-content screening/analysis (HCS/HCA) emerged as a powerful tool in the study of stem cells [55]. HCS combines automated fluorescence microscopy on multiwell plates and computational image processing methods, allowing the simultaneous evaluation of diverse cellular and molecular processes at the single-cell level [56]. Usually, a high-content screening assay includes a DNA-intercalating fluorescent stain, such as Hoechst or DAPI, used to delineate the nuclei, which is digitally segmented as a unique object identifying each cell in the image. Additionally, cytoplasm (or other compartments) can also be delineated and segmented (i.e., by a second stain in a distinct channel), allowing several morphological features of the cell to be quantitatively evaluated. Additional channels can then be used to access the expression level and subcellular localization of different proteins, by immunofluorescence (using specific antibodies). Alternatively, cell lines harboring a gene reporter of a fluorescent proteins (such as GFP) fused to a specific protein gene or downstream a specific promoter can be used to track, respectively, the subcellular localization of the fusion protein or the transcriptional activity of the given promoter, by live cell imaging [53, 57, 58].

Next, we describe all studies carried up to date that used arrayed screenings to evaluate the functional roles of genes or miRs in pluripotency, reprograming, differentiation, and Naïve/Primed interconversion.

## Screenings in pluripotency, reprograming, and differentiation

Despite the enormous potential of arrayed functional screenings based on single-cell-level primary readouts, comprehensive studies taking full advantage of such approaches, to explore the biology of human pluripotent cells, are largely lacking. Usually, the screenings carried were based on assays that use plate readers (spectrophotometers, fluorimeters, or luminometers), only allowing the measurement of a single parameter corresponding to an average response of the cell population, NCBI Assay Guidance Manual [47, 59]. The majority of the screenings described here, used cell lines harboring distinct gene reporters (e.g., GFP, luciferase, puromycin resistance) under the control of regulatory promoter regions from Oct4 or Nanog genes, which represent the most commonly used and straightforward nuclear markers associated with pluripotency [7–10].

### RNAi screenings

As described, RNA interference can be attained by several experimental approaches, but, in general, synthetic siRNA delivered by lipotransfection (LT) predominate, followed by shRNA vectors delivered by lenti- (LV) or retroviruses (RV). Given the easier (less demanding) manipulation of mESCs, as compared to hESCs, the majority of these studies were carried using mouse-derived cells. Also, given the enormous number of gene transcripts and the need to use multiple targeting molecules for each transcript (in order to discriminate specific from off-target effects), screening campaigns were usually automated to a large degree. However, some studies were carried manually, evaluating a limited set of targets. Table 1 summarizes all the RNAi screenings carried up to date. In this table, different columns describe the following: the number and general description of the set of genetic elements screened (libraries used) and the method used for introducing them in the cell; the cell model used (specie, cell line, reporter gene and biological context); the acquisition system and method used; the readout parameter(s) evaluated; the main findings; and the corresponding reference of the publication. Among the main findings, we included the number of hits (genes) identified in the screening, the number of validated hits (when provided), a representative list of hits (mainly those discussed in the manuscript of origin), and comments related to additional functional validations (such as reprograming experiments with specific genes).

#### Pluripotency/differentiation

The first study to carry an arrayed screen (in 2006) used quantitative PCR (qPCR) to manually access Oct4 gene expression levels in mESCs following the introduction of 326 RNAi plasmids, carrying cloned cDNAs (selected by subtracting M13 mESC with MEFs) transcribed by two opposing cytomegalovirus promoters [60]. Ten of the plasmids decreasing Oct4 expression (and also reducing the number of AP+ colonies to 20% of controls) coded for 8 genes, including Zfp42/Rex-1, whose knockdown induced mESC differentiation to endoderm and mesoderm.

In the same year, Ivanova et al. used LV to transduce mESCs with vectors expressing shRNA (and GFP) against 70 mouse transcript genes (downregulated in mESCs upon RA-induced differentiation). GFP+ (shRNA-expressing) cells were mixed in a 4-to-1 ratio with non-transduced cells, and after each culture passage, the ratio of GFP+/total cells was evaluated by flow cytometry [61]. Progressive decreases in these ratios, in 10 out of the 70 genes, revealed that Esrrb, Tbx3, and Tcl1, as well as other known pluripotency factors (Nanog, Oct4 and Sox2), were required for efficient self-renewal of ESCs in vitro; moreover, downregulation of these genes induced differentiation along specific lineages.

Another study manually carried used transmitted light microscopy to screen a focused esiRNA library of 1008 mouse genes encoding chromatin proteins, evaluating viability, and alterations in cell or colony morphology [62]. Strikingly, among 68 transcripts whose knockdown led to diverse phenotypes, seven coded for subunits of the Tip60-p400 complex, indicating its role in the maintenance of mESC characteristic features, likely regulating gene expression of genes marked by H3K4me3 and with Nanog-bound promoters. Using the same approach (but shRNA instead of esiRNAs), Das et al. evaluated the effects of 20 histone demethylases (HDMs), finding that Jmjd2b/Kdm4b and Jmjd2c/Kdm4c (both acting in H3K9me2/me3 and H3K36me2/me3) were essential for mESC identity and also for efficient somatic cell reprogramming [63].

All remaining RNAi screening used automated approaches, some focusing on specific sets of genes with particular molecular functions or, otherwise, based on comprehensive genome-wide libraries. Among focused screenings, Bilodeau used shRNA against 197 chromatin regulators to identify genes whose knockdown on mESCs led to decreased Oct4 staining intensity (averaged by the number of cells, as defined by Hoechst-stained nuclei), as quantified by HCS (ArrayScan HCS Reader, Cellomics) [64]. Loss of H3K9 methyltransferases, particularly SetDB1, reduced Oct4 expression, likely by de-repressing “bivalent” genes encoding developmental regulators, commonly repressed by Polycomb group proteins.

Also focusing on 312 mouse genes involved in chromatin regulation, early developmental signal transduction, and transcription, Schaniel used automated flow cytometry to quantify the percentage of Nanog-GFP+ NG4 mESC cells after RA-induced differentiation, following LT of shRNAs [65]. Forty genes were found to decrease Nanog-GFP+ % below the % observed for Oct4 knockdown, or to increase it above the knockdown of the retinoic acid receptor (RARalpha). Among the latter, members of the PBAF chromatin remodeling complex, including Smarca4/Brg1, Smarcb1/Baf47, Smarcc1/Baf155, and Smarce1/Baf57, were shown to be required for the repression of Nanog and other self-renewal genes upon differentiation.

Another focused screening used siRNAs, against 571 kinases and 253 phosphatases (each one targeted by 3 pooled siRNAs), and a mESC CGR8 cell line with a luciferase reporter under the control of a promoter specific for β-catenin-TCF/LEF binding and identified genes modulating Wnt/β-catenin signaling [66]. From all genes screened, 14 increased Wnt/β-catenin signaling when silenced, while 55 decreased it; among the latter, AURKA (Aurora kinase A), CKB (creatine kinase-brain), TEC (protein tyrosine kinase), and MTMR6 (myotubularin related protein 6, from the phosphatase library) were validated independently with the 3 separated siRNAs. Moreover, the majority of hits downregulating Wnt/β-catenin signaling were found to be interconnected in some way, as indicated by network interaction analyses.

Also focusing in kinases and phosphatases, Wang et al. screened 4801 shRNAs against 929 mouse genes and used automated spectrophotometry to sequentially quantify the reduction of AlamarBlue-AB (as a surrogate indicative of a relative cell number) and alkaline phosphatase-AP activity normalized by AB (as an indicative of pluripotency), in D3 mESCs [67]. They identified 358 shRNAs (targeting 132 genes) reducing AB and AP/AB ratios (similar to the control Oct4-shRNA), 27 of which also induced morphological changes indicating differentiation, including Nme6 and Nme7, both members of nucleoside diphosphate kinase family and found to contribute to the regulation of Oct4, Nanog, Klf4, c-Myc, telomerase, Dnmt3B, Sox2, and ERas expression.

Using a self-renewal competition assay and automated flow cytometry, similar to that carried by Ivanova et al. [61], Lee et al. evaluated 104 mouse ESC-associated phosphoregulators in the CCE mESC line, identifying the Aurka-p53 signaling pathway as a critical cell fate regulator in the maintenance and reacquisition of pluripotency [68, 69].

Another study used lipotransfected siRNAs (4 pooled siRNAs for each target) to screen 247 ESC-enriched RNA-binding proteins (RBPs) in R1 mESCs using qPCR to quantify the expression of Nanog and the markers Fgf5 and Cdx2, associated with differentiation of embryonic and extraembryonic lineages, respectively [70]. They identified six RBPs, including Krr1 and Ddx47, part of a complex mediating 18S rRNA biogenesis, which contributed to sustained protein levels of Nanog and Esrrb and to efficient iPSC reprogramming.

Another group screened 640 known and predicted genes from the ubiquitin-proteasome system in Nanog-GFP NG4 mESCs, using automated flow cytometry [72]. The screening in the presence of LIF revealed 20 genes whose knockdown lead to significant loss of mESC self-renewal (reduced % of GFP+ cells). Of these, nine were validated, including deubiquitinase Psmd14, found to be essential to pluripotency. Repeating the screening in the absence of LIF and in the presence of RA, led to the identification of 17 genes that, when silenced, led to significant upregulation of Nanog-GFP expression. Nine were further validated, including the E3 ligase Fbxw7. Authors went further showing that Psmd14 and Fbxw7 played significant opposing roles in cellular reprogramming.

Using a HCS approach, Honarpour et al. screened 1066 genes of the ubiquitin-proteasome system, using a mESC DTC26 cell line with GFP under the control of the cardiac-specific promoter of the alpha-myosin heavy chain, to identify ubiquitin system genes that repress cardiovascular tissue formation [73]. The screen uncovered the F-box protein Fbxl16 as a repressor of FLK1+ progenitor formation.

Although focused screenings allow researchers to gain insights on the roles of genes with specific molecular functions, a real comprehensive view of the complex biological processes acting on pluripotency, differentiation, and reprograming requires a genome-wide evaluation. Despite the great potential of these so-called hypothesis-free screenings, the large amount of genes to be tested translates in expensive and laborious screening campaigns. As mentioned, these screenings demand a large degree of automation and practically all of them were carried using mESCs.

One of the two first large-scale screenings to be carried involved the functional evaluation of 25,057 mouse genes in Oct4-Gip mESC carrying a Oct4-GFP reporter, lipotransfected with esiRNAs and submitted to automated flow cytometry [74]. Selected factors with the strongest positive effect on Oct4 expression belonged to different gene ontology functional classes, including protein degradation/DNA repair, signaling, chromatin modulation, and transcription regulation. Among the transcription regulators, Rtf1 and Ctr9 were both components of the Pol II-associating factor 1 complex (Paf1C), which was then shown to bind to the promoters of key pluripotency genes, contributing to the maintenance of a transcriptionally active chromatin structure.

In the same year, Hu et al. published a study that used a library with 16,683 siRNAs, which was also screened using the same Oct4-Gip mESC line and automated flow cytometry [75]. From the 148 genes whose knockdown was found to cause differentiation, Cnot3 and Trim28 were further studied and were found to co-occupy many gene promoters along with c-Myc and Zfx, but not with the core module formed by Nanog, Oct4, and Sox2, revealing an independent module associated with self-renewal.

The first study to use a HCS approach in a larger scale, functionally evaluated 10,000 genes, using lentiviral-delivered shRNA expression vectors, quantifying average cell Oct4 intensity in mESCs stained with anti-Oct4 and Hoechst [76]. In addition to most of the known pluripotency factors, the screen also revealed 12 subunits of the mediator complex (Med) and 3 of the cohesin complex (Smc1a, Smc3, and Stag2), as well as the cohesin loading factor (Nipbl). Further studies demonstrated that mediator and cohesin complexes physically and functionally connect the enhancers and core promoters of active genes in mESCs.

Using the same genome-wide library with 16,683 siRNAs, Gingold et al. carried a screening using a confocal HCS system (ImageXpress Ultra, Molecular Devices) to evaluate the percentage of Nanog-GFP+ (NG4 mESCs), following LIF removal and RA-induced differentiation, identifying 728 candidate hits with potential functional significance in pluripotency [78]. Remarkably, they found opposing effects of the transcription factors Snai1 (Snail) and Snai2 (Slug), both usually associated with an epithelial-mesenchymal transition, on Nanog expression and in Nanog-driven reprogramming, with Snai1 acting as a protein partner of Nanog co-binding and activating transcription of pluripotency-associated genes (including Lin28 and miR-290-295), and facilitating reprograming.

Of notice, Gingold et al. found an intriguing limited overlap between their hits and those from other published RNAi screens [74, 75, 77], what may derive, in part, from differences in the experimental design adopted, as noted by Subramanian et al. [79].

Strikingly, from all the siRNA screenings in pluripotent cells found in the literature, only three used human cells, revealing the overwhelming complexity associated with the manipulation of human ESCs, including transfection automation and culture.

Given the mentioned complexity in the manipulation of hESCs, one study used human NTera2D1 EC cells to screen 319 human genes, whose expression was found to be enriched in human NTera2D1 and H9 ESCs. Only the relative cell number (total Hoechst intensity per well, measured by a fluorimeter) was evaluated as a surrogate indicator of pluripotency loss [81]. Among 23 confirmed hits, some were involved in transcription (including ZIC3, NANOG, SOX2, HMGA1, POU5F1, and NR5A2) or cell cycle progression and apoptosis (including RBBP9, BIRC5, and CDC2).

The other two studies used H1 hESCs with a GFP reporter under the promoter control of the POU5F1/Oct4 [82] or Nanog genes [83] and evaluated the percentage of GFP+ cells among total cells (i.e., number of Hoechst stained nuclei), using an automated confocal fluorescence microscope. By screening 21,121 siRNAs pools, Chia et al. identified 566 genes increasing the percentage of Oct4+ cells. Enrichment analysis, for gene ontology (GO) terms or Reactome pathways, identified transcription factors and translation factors as the main group of genes enriched, including PRDM14, which was further shown to promote reprograming, cooperating with other key transcription factors such as OCT4, NANOG, and SOX2 [82].

In turn, Gonzales screened 4558 human genes with siRNAs in H1 hESCs cultured under 5 differentiation media: with addition of retinoic acid, lacking bFGF and TGF-b or with the addition of inhibitors for TGF-b, MEK, or PI3K signaling [83]. As a result of the combined analyses of all 5 screenings, they found genes acting in processes centrally important in what they called pluripotent state dissolution (PSD), including histone acetylation, chromatin remodeling, RNA splicing, signaling pathways, and more importantly, a strong and specific enrichment of cell-cycle genes involved in DNA replication and G2 phase progression. More specifically, high levels of Cyclin B1 in G2 phase were associated with the prevention of PSD. Overall, these findings stablished a strong basis for the known relationship between G2 cell-cycle phase and pluripotency, and with G1 phase and differentiation.

Of notice, to our knowledge, only a single study carried an arrayed gain-of-function screen in the context of pluripotency. In this study, Abujarour et al. transfected plasmid expression vectors of a library containing 30,892 cloned cDNAs from mouse and human, on mouse P19 embryonal carcinoma cells harboring a Nanog-luciferase reporter [84]. Among confirmed hits, 90 activated the luciferase reporter (including Timp2, Hig2, and Mki67ip) and 14 inhibited it (including PU.1/Spi1, Prkaca, and Jun). Moreover, the mentioned hits also promoted self-renewal or differentiation when expressed in mESCs.

#### Naïve-primed transition

As mentioned, mESCs derived from the ICM of pre-implantation embryos are in a “Naïve” state of pluripotency that depends on LIF-JAK-STAT3 (and BMP4) signaling to inhibit differentiation and promote survival and self-renewal [17, 21], while EpiSCs derived from the epiblast post-implantation are in a Primed state that depends on FGF2-ERK (and Activin) signaling for self-renewal [19, 20, 22].

Classically, the use of two small inhibitory molecules (2i) of the MEK/ERK (PD0325901) and GSK3β (CHIR99021) pathways allow Naïve mESCs to be cultured in the absence of LIF and BMP4, characterizing what is called the “Ground State of Pluripotency” [85]. In contrast, Primed EpiSCs cannot be cultured in 2i media, given their dependence on FGF-ERK signaling for self-renewal. Mouse ESCs and EpiSCs are interconvertible, however, while the change from Naïve to Primed state is easily obtained in culture (e.g., by removing 2i or by substituting LIF with FGF); the reverse process depends on extensive epigenetic reprogramming [24, 25].

Given that ESCs in the Naïve state are endowed with the potential to differentiate to all germ layers in an unbiased manner [17] and that hESCs are considered to be in a Primed state and, thus, more similar to mouse EpiSCs than to mESCs [23], understanding which genes control pluripotency and differentiation in Naïve and Primed states bears important implications in regenerative medicine. Four studies carried screenings in this context (all using mouse cells). Three of them evaluated which genes had a role during Naïve to Primed transition, evaluating cultured Naïve mESCs following removal of 2i and/or LIF [71, 77, 86], while one study carried the screening using Primed EpiSCs [80].

The first screening was based on automated flow cytometry and used a mESC line (Rex1GFPd2) engineered to express an unstable version of GFP from the endogenous rex1/zfp42 gene locus, a marker of the Naïve pluripotency “ground state” [77]. Rex1GFPd2 ESCs were kept in 2i media to maintain their Naïve state, transfected with siRNA pools against 16,873 mouse genes and, then, had the 2i media removed to drive the conversion to the Primed state (characterized by the loss of Rex1-GFP expression). In addition to the control siRNAs for fgf4 and gsk3b, 792 siRNAs delayed the loss of GFP expression above the stipulated cutoff. Following two secondary validation screens, with the same cell and an additional Oct4-GFP reporter mESC, 316 genes where commonly identified. Interestingly, additional screening of these 316 genes with the removal of only one of the inhibitors allowed further functional dissection of the genes, revealing those acting through independent signaling pathways (MEK or GSK) or through shared mechanisms.

The second and also comprehensive study screened the druggable genome and all transcription factors (respectively, 8296 and 1640 siRNAs against mouse genes). Mouse Oct4-Gip ESCs (carrying an Oct4-GFP-puromycin resistance reporter construct) were kept in the Naïve state (by culturing them in 2i media), and following lipotransfection with the siRNAs, cells were cultured in the absence of 2i to drive conversion into the Primed state. Finally, media was substituted by 2i media again and the relative number of surviving puromycin-resistant (Oct4+) cells was quantified by automated fluorimetry (Alamar Blue Intensity/Well). From 46 primary hits, 28 were validated, including Tsc1/2 and Flcn (Folliculin), whose knockdown prevented transition to the committed primed state (i.e., increased number of puromycin-resistant Oct4+ cells). Functionally, knockdown of Tsc1 or Tsc2 and Flcn induces nuclear accumulation of the transcription factor Tfe3, thus restricting the transcription of pluripotency factors such as Esrrb and other targets, impairing exit from pluripotency [86].

The third study in the context of Naïve-Primed transition used a HCS assay to screen siRNAs against 356 putative RBPs in mESCs with mCherry knocked into the mir-290∼295 locus (a Naïve marker) and eGFP knocked into the mir-302∼367 locus (an EpiSCs marker). When Naïve mESCs are allowed to differentiate into Primed cells through the removal of LIF and 2i media, mir-302 is turned on and mir-290 is turned off later on [71]. Following removal of 2i and LIF and transfection with siRNAs, knockdown of 49 genes significantly changed the expression levels of miR-290-mCherry or miR-302-eGFP (total stained area, normalized by the Hoechst-stained area) and did not negatively affect cell proliferation/viability (relative reduction of Hoechst staining). Interestingly, knockdown of 34 genes led to a reduction in miR-302-eGFP expression; however, knockdown of only one gene, Ilf2 (NF45), increased miR-302-eGFP expression and, also, impaired ESC proliferation and dysregulated lineage differentiation, suggesting a role on the inhibition of premature differentiation out of the Naïve state.

The study that explored pluripotency in Primed EpiSCs was also the last genome-wide RNAi screening using mouse-derived pluripotent cells published up to date and was carried by Ding et al., using automated flow cytometry to evaluate the effects of esiRNA-mediated knockdown of 12,348 mouse genes in the Oct4-GFP EpiSCs line OE7, maintained in medium supplemented with Activin A and Fgf2 [80]. As result, Ding identified 467 hits downregulating Oct4 expression in EpiSCs, which included many of the core pluripotency factors, as well as other components previously identified in screenings with Naïve mESCs. On the other hand, remarkably in contrast to other reported RNAi screens in Naïve mESCs, where knockdowns upregulating Oct4 are rarely seen, their screen identified 149 genes whose knockdown significantly upregulated Oct4 expression in EpiSCs, indicating that Oct4 would be under active repressive control in EpiSCs.

#### Reprograming

Although many of the above described screenings, evaluating the effects of gene knockdown on pluripotency/differentiation, also carried further functional evaluation of selected hits in the context of iPSC reprograming, only four studies directly evaluated gene knockdown effects on reprograming. Of notice, all of these screenings were based on microscopy, allowing them to identify iPSC colonies. To further support the pluripotency of the observed colonies, these studies used different strategies. For instance, two siRNA screenings used a fluorescent substrate of alkaline phosphatase (AP) to measure its activity on colonies, along with DAPI nuclear co-staining, \allowing colony AP+ area to be quantified [87, 88]. A different strategy takes advantage of MEFs derived from mice carrying an Oct4-GFP reporter gene [89]. This kind of strategy may reduce or completely eliminate the need for fixation and staining; thus, real-time live-imaging can be used, allowing the appearance of GFP+ colonies to be tracked along the reprograming process, until colonies are counted in the chosen day(s). Of notice, choosing a different day for counting colonies extends the ability to identify hits affecting reprograming with distinct kinetics [90–92].

Another important technical aspect of reprograming screenings is the choice of a reprogramming system (vectors and delivery method). In this sense, systems in which transgenes are independently introduced into somatic cells by retroviruses or lentiviruses are relatively inefficient and usually result in a large variation in the reprograming kinetics. In turn, systems employing inducible (e.g., by doxycycline) cassettes with all pluripotency factors (OSKM) not only increase efficiency but also synchronize the process, allowing reprograming mechanisms to be dissected, regarding the distinct phases of reprogramming [88]. With that in mind, the majority of the studies reported in this review (siRNA or miRNA) adopted the use of vectors carrying all reprograming factors transcribed in tandem from a single cassette, many of them using Dox-inducible systems, thus allowing for a more controlled expression of the factors in the cells (see Tables 1 and 2 for references).

Many of the reported studies used primary MEFs. In this regard, their limited proliferative potential usually restricts the execution of genome-wide screens, given the larger requirement in total cell numbers [87]; however, the use of cell lines with longer lifespan (such as BJ fibroblasts) eliminates this specific restriction, allowing large-scale screenings to be carried [93]. Importantly, while primary screens on reprograming allow the identification of genes promoting or repressing the appearance of early colonies with primary characteristics of iPSCs (iPSC-like), the final confirmation that these identified conditions allow the generation of truly fully reprogramed stable transgene independent iPSCs usually depend on extended periods of culture with additional characterizations using a panel of markers that may include downregulation of the fibroblasts CD13 marker and upregulation of the cell surface markers of pluripotency, such as SSEA4 and TRA-1-60, TRA-1-81 [94]. Moreover, functional characterizations may include confirmation of retroviral transgene silencing, differentiation potential into all three germ layers in vitro, in embryoid bodies or in vivo, on teratoma formation assays [95] and, ultimately, on chimeric mice generated by aggregation of iPSCs with early embryos [87, 89, 93].

The first screening we describe, carried by Samavarchi-Tehrani et al., evaluated the effect of siRNA-mediated knockdown of 4010 mouse genes (including all genes in categories, such as signaling, transcription factors, and chromatin regulators), using a Dox-Inducible OKMS cassette to reprogram MEFs [87]. Using an HCS system (InCell Analyzer 1000, GE), they quantified the total iPSC colony area (overlap between AP and DAPI staining), identifying that knockdown of genes associated with mesenchymal-epithelial transition (Cdh1, Par3, and Crb3) and the BMP pathway (Smad4, BMPRII, and the BMP type I receptor, ALK3) strongly suppressed the appearance of AP-positive reprogrammed colonies. Moreover, they showed that these effects were linked to the BMP-dependent induction of miR-205 and miR-200 family, key regulators of MET during the initiation phase.

In a distinct setting, Sakurai et al. used an automated confocal fluorescence microscope to evaluate 734 mouse kinase genes (targeted by 3686 screened shRNA) during reprograming (retroviral delivery of OSKM factors) of MEFs carrying an Oct4-GFP reporter gene [89]. Interestingly, 59 kinases were identified as barriers to iPSC generation and knockdown of the serine/threonine kinases TESK1 or LIMK2 was shown to promote mesenchymal-epithelial transition, decreasing COFILIN phosphorylation and disrupting actin filament structures during reprogramming.

Using the same approach used by Samavarchi-Tehrani [87], Hirsch et al. evaluated 652 epigenetic regulators, identifying components of the SAGA histone acetyltransferase complex (including Trrap, Ccdc101, Taf12, and Gcn5) as critical regulators of reprogramming initiation. In particular, Gcn5 strongly associates with Myc, and upon reprogramming, they activate a distinct alternative pluripotency-associated splicing network [88].

The last and most comprehensive study in the context of reprograming carried a whole-genome siRNA screening (21,121 human genes) during OSKM-mediated reprograming of BJ human fibroblast, using a confocal HCS system (ImageXpress Ultra, Molecular Devices) to quantify the stained TRA-1-60+ area, normalized by the number of cells [93]. Strikingly, combinatorial knockdown of five identified repressors (SMAD3, ZMYM2, SFRS11, SAE1, and ESET) synergistically led to a highly efficient, almost deterministic, reprogramming (85%). Mechanistically, SFRS11 was found to regulate exon skipping and mutually exclusive splicing of ZNF207 transcripts and its knockdown, during reprograming, promoted rapid acquisition of pluripotency-specific spliced forms.

### microRNA screenings

Almost all (except one) functional screenings exploring miRNA roles on the biology of mouse pluripotent cells were carried using synthetic miR mimics introduced by lipotransfection (Table 2). Among these, Wang and colleagues manually screened a library of 266 synthetic mouse miRNAs in mESC knockout for DGCR8 (largely deficient in the processing of miRNAs), identifying several miRNAs able to recover the proliferation defect of these cells (accumulation in the G1 phase of the cell cycle), as spectrophotometrically evaluated using the MTT assay [96]. Among the hits, members of the miR-290 family (including miR-291a-3p, miR-294, and miR-295) were found to promote the transition of cells from G1 to S phase, being referred to as ES cell-specific cell cycle-regulating (ESCC) miRNAs.

Using the same DGCR8 KO mESCs, Ma et al. manually screened 40 microRNAs, evaluating their effects on differentiation through the use of colony formation assays, AP staining, cell cycle analysis, gene expression pattern, and Oct4 staining. A total of 14 miRs were identified, of which miR-27a and miR-24 were transcriptionally repressed by c-Myc and directly targeted pluripotency-associated factors (Oct4 and Foxo1) and signal transducers (gp130 and Smads) [97].

Also using Dgcr8−/− mESCs, Gu et al. manually evaluated the effects of 21 miR during Naïve to epiblast-like cell (EpiLC) transition, carrying qRT-PCR analysis of Naïve markers (Rex1 and Klf2) and post-implantation epiblast marker Fgf5. Surprisingly, they found that the pluripotency-associated miR-290/302 family facilitated the exit of Naïve pluripotency, in part by repressing Akt1 and promoting the activity of MEK pathway [98].

Despite the claimed advantages of DGCR8 KO mESC in functional miR screenings, DGCR8 forms an alternative complex with the exosome (the major RNA decay machinery involved in processing and degradation of RNAs) and acts in the maturation of snoRNAs and in the degradation of telomerase RNA, what may functionally impact the miR effects observed in these screenings [99]. Furthermore, in a later work carried by Wang, he found that let-7 and miRs-26a, 99b, 193, 199a-5p, and 218 were able to downregulate the AP activity on Dgcr8 knockout mESCs, but not on wild-type cells (likely by the antagonizing effect of endogenous miRs of the miR-294/miR-302 family), underlining important differences of this DGCR8 KO model [100].

Other three studies screened for miRNAs were able to promote OSKM-mediated reprograming of MEFs into iPSCs. One study used an Oct4-GFP reporter MEF cell line (in which an IRES-PuroGFP cassette was targeted to the 3′-UTR of the Oct4 locus) to screen a set of 52 candidates of mESC-specific miRNAs (expressed using a piggybac miR vector introduced by electroporation). By using puromycin selection and flow cytometric quantitation of GFP+ cells, Lu et al. found that miR-25 enhanced iPSC generation by targeting two ubiquitin ligases, Wwp2 and Fbxw7, proposed to regulate Oct4, c-Myc, and Klf5 [101].

Two more comprehensive screenings reprogramed MEFs (with a GFP reporter under the control of an Oct4-driven promoter) using OSK factors (but not c-Myc), evaluating the effect of a total of 379 or 570 transfected mouse miRNAs mimics on GFP+ colony counts, either by semi-automated immunofluorescence microscopy or by HCS (using an InCell Analyzer 2000 from GE), respectively [90, 102]. In the former work, considering a fourfold induction in the number of GFP+ reprograming events in the eighth day evaluated, Pfaff et al. identified 19 miRNAs (corresponding to 27 distinct mature miRNA variants). Of these, 14 were previously unreported, including members of the miR-130/301/721 family, which would act by targeting the homeobox transcription factor Meox2 (also known as Gax), as Meox2-specific silencing mimicked miRNA effects [90].

In turn, Judson found 16 miRNA mimics which enhanced reprograming in two independent screenings, including previously identified ESCC miRNAs of the miR-302-miR-294 family and the novel miR-181 family. Inhibition of the latter by anti-miRs diminished iPSC colony formation, indicating that OSK acts, in part, by inducing endogenous miR-181. Moreover, the group used siRNA to functionally evaluate the effect on reprograming of 58 predicted target transcripts of miR-181 and 56 predicted targets of miR-294, which were known to be expressed in MEFs, iPSCs, or ESCs and whose expression in different cell types was previously shown to be inverse correlated to the levels of the corresponding targeting miRNAs. Knockdown of 8 targets (from each miRNA) enhanced reprograming, indicating that the effects of these miRs would be mediated, at least in part, by the post-transcriptional regulation of these targets [102].

While in his initial paper, Pfaff focused in miRs promoting reprograming [90, 91]; in a more recent paper, they revisited the same screening, but focusing in miRs hampering the reprograming process. A total of 14 miRNAs reduced the number of GFP+ colonies by fourfold, of which the miRNA-212/132 family was shown to hamper reprograming, at least in part, by targeting the epigenetic remodeling factors p300 and Jarid1a, as their knockdown (using siRNA) recapitulated miRNA effects [92].

The largest study exploring miR effects on mESCs was carried by Colas and McKeithan, which evaluated the effects of 875 human miRs on mesoderm differentiation, using a HCS system (InCell Analyzer 1000, GE) to quantify the integrated fluorescence of a cardiac-specific reporter eGFP gene under the control of the Myh6 promoter [103, 104]. As a result, they found that miRs of the let-7 and miR-18 families promote mesoderm differentiation (both, cardiomyocyte and endothelial) at the expense of endoderm, by targeting Acvr1b and Smad2, respectively, and inhibiting Nodal signaling.

Although the aforementioned screens helped to pinpoint important miRNA functions in the regulation of reprograming, self-renewal, pluripotency and differentiation, they were all carried using mouse cells. Moreover, these studies were limited in the number of phenotypic readouts evaluated. Indeed, only few of these screenings mention the use of high-content screening; however, the true information content lags behind, as only one or two image-based features were measured. Usually, per-cell readouts were summarized as a single mean value or percentage, obscuring subtle changes that are present only within certain subpopulations of cells [105]. Such approaches are not able to detect unanticipated effects on cell physiology, such as those manifested as morphological and phenotypic changes at the cellular level, losing valuable information [106].

With that in mind, we recently carried the first focused high-content microRNA functional screening (using an ImageXpress^Micro^ HCS system Molecular Devices), based on two of the most widely studied human pluripotent model cell lines, H1 hESCs and NTera-2 ECCs. For this, cells were transfected with 31 miRNA mimics, found to be differentially expressed between pluripotent and differentiating cells [12, 107], cultured, and stained with Hoechst/CellMask Blue, to segment the nuclear and cytoplasmic compartments, and with fluorescent-conjugated antibodies against OCT4 and Cyclin B1, as surrogate indicators of pluripotency and cell cycle status. Several cellular and nuclear morphological features, as well as intensity measurements of both proteins in these nuclear compartments, allowed us to generate a multiparametric phenotypic profile describing the effects of each miRNA on these cells. By identifying transcripts commonly targeted by microRNAs inducing similar multiparametric phenotypic profiles (as revealed by hierarchical clustering of the multiparametric profiles and in silico target prediction), we were able to identify signaling pathways and biological processes involved in pluripotency and differentiation, which were likely to be post-transcriptionally modulated by the corresponding microRNAs from the groups identified. Specifically, we found that miR-363 contributes to pluripotency maintenance, at least in part, by targeting NOTCH1 and PSEN1 and inhibiting Notch-induced differentiation; a mechanism that could be implicated in Naïve and Primed pluripotent states. Integration of this type of data with similar data obtained from siRNA screenings (using the same HCS assay) could provide a large-scale functional approach to identify and validate microRNA-mediated regulatory mechanisms controlling pluripotency and differentiation. Although this review focused on genetic screenings relying in the use of siRNAs, miR mimics or cDNA expression vectors, one study evaluated the effects of extra celular matrix proteins on hESCs submitted to endodermal differentiation. 1280 combinations of collagens, fibronectin, laminin and vitronectin were deposited in microarrays and hESCs were then seeded on top of slides. Intensity of the endoderm marker SOX17 (normalized by Hoechst) was used as an indicative of differentiation. Authors found that fibronectin and vitronectin promoted differentiation trough interactions with integrins ITGA5 and ITGAV, respectively, as shRNA-mediated KD of both integrins on hESCs disrupted differentiation [108].

## Limitations and future perspectives

### Design-related issues on gain- or loss-of-function tools

Although siRNAs and shRNAs are designed using stringent algorithms that try to increase knockdown efficacy and specificity (by avoiding additional target sites in the transcriptome), the shared silencing mechanism shared with microRNAs limits the specificity that can be attained. More specifically, in addition to the specific cleavage of a given site on the target transcript (fully complementary to the siRNA), the siRNA may have broad unspecific off-target “miR-like” effects, if its 5′ region has complementarity to other transcripts, by mechanisms similar to those mediated by the 5′ seed region of microRNAs [39].

Moreover, given that siRNAs, shRNAs, and miRNAs are double-stranded RNA molecules, although the design of the passenger strand sequence can lead to the preferential loading of the desired guide strand in the RISC complex, this may not be always the case. In the case of synthetic molecules, even with the presence of chemical modifications in the passenger or guide strands (to destabilize or stabilize them, respectively), it is not 100% guaranteed that only the desired strand will be loaded into RISC. As an example, synthetic miR mimics from Ambion (Life Technologies), named “Pre-mir™ miRNA Precursors” (which are not real hairpin pre-miRNAs), are double-stranded molecules composed of the desired mature miR guide strand and a chemically modified (destabilized) passenger strand [109]. These molecules, according to the manufacturer, are designed and modified to ensure that the correct strand representing the desired mature miRNA is taken up into RISC; however, a second generation of miRNA mimics, named “mirVana™ miRNA Mimics,” from the same manufacturer, is allegedly more specific than their predecessors, due to new proprietary chemical modifications inactivating the passenger strand more efficiently.

These modifications, on the one hand, allow the effect of only one of the strands of the mature miR to be studied; however, both strands can mediate biological effects (depending on the cellular context). Thus, on the other hand, such modification prevents the simultaneous functional evaluation of both strands of mature miR [110].

A different design strategy that claims to completely abolish the possible effects of the passenger strand involves the use of two short complementary passenger strands (instead of only one) modified with locked nucleic acids (LNA) to stabilize their interaction with the guide strand (miRCURY LNA miRNA Inhibitors, Exiqon/Qiagen); however, whether LNA may limit the release of the guide strand in some cases is not well documented.

In the case of siRNAs and shRNAs, distinct sequence designs, targeting different regions of a given transcript, can be used to check if a given phenotype is specifically caused by knockdown of the target transcript and not by unspecific off-target effects of guide or passenger strands.

To minimize unspecific off-target effects of a given siRNA, its concentration should be reduced to a minimum, so that the miR-like effects are minimized. In that sense, esiRNAs have a remarkable advantage, as they correspond to pools of siRNAs generated in vitro from cleavage (using *Escherichia coli* RNase III) of long double-stranded RNA (dsRNA), originated by annealing of strands bi-directionally transcribed, in vitro, from a large (hundreds of bps) cDNA region of the target transcript. As a result, each siRNA of the esiRNA pool is present at a very low concentration, minimizing off-target effects, while the combined total amount of siRNAs targeting the transcript allows an efficient knockdown. Though less variable in their performance in gene silencing, esiRNAs would be expected to be more susceptible to cross-silencing of homologous genes, depending on the region used for their generation; however, there are available algorithms that can help in the selection of the region to be used, based on the highest possible number of highly effective siRNA and the minimum potential to cross-silence homologous gene transcripts [111], as used by the Mission esiRNAs from Sigma.

Another alternative to reduce off-target effects is to use defined pools of a limited number of siRNAs (usually 3 or 4) designed independently, a strategy adopted by some manufactures such as Dharmacon/GE Life Sciences, which offer the siGENOME and TARGETplus libraries as a “SMARTpool” or 4 individual siRNA reagents. This allows an efficient silencing and slightly reduces the unspecific effects. Moreover, once the pool is identified as a hit driving a given phenotypic effect, each of the siRNAs in the pool can be evaluated independently, in order to verify that the observed effect is not an unspecific effect mediated by one of the siRNAs in the pool.

A completely different strategy to knockdown RNA levels in a cell exploits the endonucleolytic cleavage of a RNA strand, mediated by RNase H1, when it is complexed to a DNA strand. One design, adopted by Exiqon/Qiagen (Antisense LNA GapmeRs), consists of a single-stranded 16mer oligonucleotide containing a central DNA portion flanked by LNA modified regions. While the LNA increases target affinity and confer nuclease resistance, the unmodified central DNA hybridized region allows recognition and cleavage of the RNA strand by RNase H [112]. Of notice, this method does not suffer from the miR-like RISC-mediated off-target effect and, importantly, can knockdown RNA targets irrespectively of the subcellular location, allowing lncRNAs to be targeted in the nucleus (were the RNAi machinery is not available).

### Transient versus constitutive effects

There are several limitations in the study of microRNA functions, depending on the adopted experimental approach. For instance, synthetic miR molecules may trigger double-stranded RNA sensor pathway and passenger strands, depending on their design, may have an effect that may be cell type-specific [113]. Moreover, the concentration of synthetic molecules, or the expression level of the specific vector used, may result in a completely different set of transcript targets being modulated and, thus, a completely different functional outcome [114].

While synthetic miRs (or their inhibitors) or siRNA exert their function in a transient manner, the use of miR/shRNA vectors allow a strong and constitutive expression. As a result, while the first evaluates the effect of a relatively milder modulation at a given moment on a given process, the latter evaluates the effect of a stronger modulation throughout the entire given process [115]. Moreover, while siRNA or shRNAs have a single target, miRs have several targets with distinct binding specificities; thus, while transient modulation may affect a restricted target set for a shorter period, constitutive modulation may affect several additional targets by longer periods, in a way that additional secondary biological effects may occur and prevail. As an example, Yang and collaborators showed that miR-29a depletion by a synthetic anti-miR inhibitor would enhance MEF reprogramming efficiency [116], a result in line with a screening where transfected mimics for all members of the miR-29 family hampered reprogramming [90]. In contrast, Guo et al. showed that retroviral-mediated miR-29b expression throughout the process promoted reprogramming, an effect that would stem from the global demethylation resultant from downregulation of targeted DNA methyltransferases Dnmt3a and Dnmt3b [117]. Moreover, in a systematic RNAi screening in MEF reprogramming (similar to the one adopted by Pfaff et.al.), siRNAs against DNMT3a and DNMT3a did not affect colony formation, indicating that transient reduction of DNMT3a/b (directly by siRNAs or by miR-29 mimics) would not promote reprogramming [87]. Based on these conflicting results, and also based on a work from our group, which identified Tet1, 2, and 3 (components of active DNA demethylation) as targets of miR-29 [118], we further explored miR-29’s role in reprograming using synthetic miRs and anti-miRs. We showed that by targeting Tet1, 2, and 3 and Gsk3-beta (the phosphatase involved in B-catenin degradation), miR-29a hampers reprograming, likely by interfering with hydroximethylation and by activating Wnt signaling, respectively [119].

### Model cell lines

Several human or mouse ESC lines bearing fluorescent protein in fusion with distinct proteins or as reporters, have been developed and can be used in image-based screenings. As the field advances, cells with distinct fluorescent reporters may eliminate the need for fixation or staining, allowing the execution of live-imaging experiments, with the dynamic acquisition of multiple time points (time-lapse) and, in more complex settings, cell-tracking.

Importantly, there are commercially available cell lines that can be used for the generation of tailored models. For instance, a H9 hESC Cre-LoxP line, harboring a double loxP cassette into a silencing-resistant genome locus, allows a sustained transgene expression during stem cell expansion and differentiation to all three germ layers, both in vitro and in vivo. More importantly, by transducing the cell-permeable Cre protein, along with a targeting vector that contains the same loxP sites, the integrated cassette can be easily and specifically replaced by different constructs, for example, driving lineage-specific expression of reporter genes [120].

More recently, Harikumar et al. generated a mESC library with over 200 endogenously tagged fluorescent fusion proteins, using non-directed retroviral integration of an YFP/Cherry exon. They showed that the library can be used for imaging-based techniques to track proteins in living cells, screen for pluripotency-related factors, identify heterogeneously expressing proteins, measure the dynamics of endogenously labeled proteins, track proteins recruited to sites of DNA damage, etc. [121].

Most RNAi screens focus on the coding genome, but an increasing number of CRISPR screens targeting noncoding genomic regions continue to emerge, what will likely bring novel findings to the field [122].

One drawback of CRISPR/Cas9 based techniques is that it may produce heterozygous and homozygous knockout cells, in a way that phenotypes can be missed or variability can arise from mutations in the untargeted allele, hampering a straightforward study of recessive genes [123]. Haploid ESCs have been recently derived from several species, circumventing these limitations, impacting profoundly the field of genetic screening [124, 125]. Impressively, by using AN3-12 feeder-free haploid mESC and a combination of strategies, allowing unbiased genome-wide insertional mutagenesis, a biobank of 100,000 individual haploid mESC lines carrying genetically barcoded, conditional, and reversible mutations targeting 16,970 genes was recently established and made available to the public (www.haplobank.at), overcoming clonal variance by permitting functional evaluation in sister cells [126]. Although this Haplobank is unique, given its characterization and availability, its manipulation at a larger scale would require complex automated cultivation procedures.

### Advanced computational methods

As described, high-content screening (HCS) allows the simultaneous evaluation of several “single-cell-level primary readouts,” including measurements of cell morphology and expression levels of selected markers on subcellular compartments. Despite the great potential of HCS approaches, there are several computational challenges when dealing with the enormous number of cells and images in a single screening. First of all, quantitation of the phenotypical features of a given cell requires a proper segmentation of the cell and its subcellular components into digital objects. Commercial and open-source tools, capable of carrying all image processing steps for automated segmentation, have allowed the HCS field to evolve [127, 128]. It is worth mentioning that CellProfiler allows image sets from whole screening to be systematically processed using a very friendly graphical user interface based on modular pipelines, allowing segmentation and quantitation of 2D images [129] or 3D image sets [130]. While these tools usually require an extensive manual input to tune all the parameters for a given assay (cell type, stains, etc.), tools that implement machine learning methods, such as ilastik, allow users to iteratively identify background and distinct cell types (or subcellular compartments) through brush strokes directly on the image, in order to train the algorithm to automatically segment the selected objects in all remaining images [131].

Once these object features are quantified, they can be merged into a multiparametric phenotypic profile (in the form of a numerical vector) that describes a single cell. The effect of a given siRNA (or miRNA) at the population level can be summarized by averaging the multiparametric phenotypic profiles of all cells in a given plate-well (as described in our miRNA screening), giving important insights into the function of genes and miRs. However, this approach does not allow specific cell phenotypes to be identified and have their population frequency calculated. In the case of screenings evaluating differentiation of pluripotent stem cells, this particularly limits the amount of information that can be derived from such studies.

Two main approaches can be used to classify or group cells with similar phenotypes, supervised, and unsupervised. Unsupervised approaches are based on clustering algorithms, which group unlabeled cells based on the similarity of their multiparametric phenotypic profiles [132–134]. In turn, supervised approaches are mainly based on machine learning algorithms, where a human specialist assigns a reduced set of cells with the desired phenotypes to specific classes, in order to train the algorithm, which can then automatically classify all cells in the screening. It is worth mentioning that CellProfiler Analyst integrates with CellProfiler, easily allowing users to carry supervised classification based on distinct machine learning algorithms [135].

Instead of manual annotation, novelty detection methods, implemented in open-source suites such as CellCognition Explorer, can be used to automatically identify unanticipated rare cell phenotypes. Importantly, a complementary deep learning algorithm in this suite can be used to automatically extract numerical feature sets, independently of accurate object segmentations, requiring only the center coordinates of the cell [136].

Also worth of mentioning, KNIME is another open-source platform that offers a user friendly graphical interface with an extensive library of modules (carrying out the most diverse operations, from image processing and quantitation to complex analysis) that can be joined into a pipeline, enabling complex analytical processes to be carried [137].

## Conclusions

Arrayed screenings are a powerful tool to functionally explore the roles of genes and microRNAs in the biology of pluripotent stem cells and in processes, such as reprograming, differentiation, and transition between Naïve and Primed states. Ultimately, the integration of data derived from arrayed screenings [138] will lead to major advances in the field of stem cell research and therapy.

## Additional file

Additional file 1: **Table S1.** Examples of commercially available arrayed mouse and human genetic libraries. (DOCX 49 kb)

## Abbreviations

ATRA: All-trans retinoic acid
ECCs: Embryonal carcinoma cells
EpiLCs: Epiblast-like cells
ESCC: ES cell-specific cell cycle regulating
esiRNA: Endoribonuclease-prepared small interfering RNA
HCS: High-content screening
hESCs: Embryonic stem cells
ICM: Inner cell mass
iPSCs: Induced pluripotent stem cells
miR or miRNA: microRNA
NT2: NTera-2
OSKM: OCT4, SOX2, KLF4, and c-MYC
PRC1: Polycomb repressive complexes 1
pre-miRs: Precursor hairpin miRs
pri-miRs: Primary miRs
RISC: RNA-induced silencing complex
siRNA: Small interfering RNA
TFs: Transcription factors
