# Supplementary material for: Arrayed functional genetic screenings in pluripotency reprogramming and differentiation
Source: Stem Cell Res Ther. 2019 Jan 11;10:24. doi: 10.1186/s13287-018-1124-6 (PMC6330485; doi:10.1186/s13287-018-1124-6)
Supplement: Supplementary file 1 — Table S1. Examples of commercially available arrayed mouse and human genetic libraries. (DOCX 49 kb) [file 13287_2018_1124_MOESM1_ESM.docx]

Additional file 1: Table S1. Examples of commercially available arrayed mouse and human genetic libraries.

| Library Name | Type (No targets) | Characteristics | Company (Catalog No) |
| --- | --- | --- | --- |
| Silencer™ Select Human Genome siRNA Library V4 | Synthetic (21,584) | 3 individual siRNAs | Ambion/Thermo Fisher (4397926) |
| Human mirVana™ miRNA Library | Synthetic (NA) | every human microRNA in miRBase v.21.0 | Ambion/Thermo Fisher (NA) |
| Human mirVana™ miRNA Library | Synthetic (NA) | every mouse microRNA in miRBase v.21.0 | Ambion/Thermo Fisher (NA) |
| Human siGENOME siRNA Library - Genome | Synthetic (~18,000, NM_ RefSeqs) | SMARTpool or 4 individual siRNA reagents. | Dharmacon/GE Life Sciences (G-005005-025 or GU-005005-025, Set of 4) |
| Mouse siGENOME siRNA Library - Genome | Synthetic (~19,000 NM_ RefSeqs) | SMARTpool or 4 individual siRNAs. | Dharmacon/GE Life Sciences (G-015005-025 or GU-015005-05) |
| Human ON-TARGETplus siRNA Library - Genome | Synthetic (~18,000, NM_ RefSeqs) | SMARTpool or 4 individual siRNAs.  Designed and modified for greater specificity. | Dharmacon/GE Life Sciences (G-105005-025 or GU-105005-025, Set of 4) |
| Human Lincode siRNA Library - NR lncRNA RefSeq v65 | Synthetic (3430 NR lncRNA) | SMARTpool or 4 individual siRNA reagents. | Dharmacon/GE Life Sciences (G-301005-025 or GU-301005-025) |
| Mouse Lincode siRNA Library - NR lncRNA RefSeq v65 | Synthetic (1997 NR lncRNA) | SMARTpool or 4 individual siRNA reagents. | Dharmacon/GE Life Sciences (G-311005-025 or GU-311005-025) |
| Human miRIDIAN miRNA Mimic Library 19.0 + 21.0 Supp. | Synthetic (2603) | every human microRNA in miRBase v.21.0 | Dharmacon/GE Life Sciences (CS-001040-025) |
| Mouse miRIDIAN miRNA Mimic Library 19.0 + 21.0 Supp. | Synthetic (1939) | every mouse microRNA in miRBase v.21.0 | Dharmacon/GE Life Sciences (CS-002040-025) |
| Human miRIDIAN miRNA Hairpin Inhibitor Library 19.0 + 21.0 Supp. | Synthetic (2609) | every human microRNA in miRBase v.21.0 | Dharmacon/GE Life Sciences (IH-001040-025) |
| Mouse miRIDIAN miRNA Hairpin Inhibitor Library 19.0 + 21.0 Supp. | Synthetic (1938) | every mouse microRNA in miRBase v.21.0 | Dharmacon/GE Life Sciences (IH-002040-025) |
| Human MISSION esiRNA whole genome library | Produced In vitro (16,744) | Endoribonuclease-prepared siRNAs | Sigma-Aldrich/Merck (NA) |
| Mouse MISSION esiRNA whole genome library | Produced In vitro (14,068) | Endoribonuclease-prepared siRNAs | Sigma-Aldrich/Merck (NA) |
| Human long non-coding RNA MISSION esiRNAs library | Produced In vitro (1,761) | Endoribonuclease-prepared siRNAs | Sigma-Aldrich/Merck (NA) |
| Mouse long non-coding RNA MISSION esiRNAs library | Produced In vitro (643) | Endoribonuclease-prepared siRNAs | Sigma-Aldrich/Merck (NA) |
| Human MISSION miRNA Mimic Library, miRBase version 21 library | Synthetic (2,754 miRNA mimics) | every human microRNA in miRBase v.21.0 | Sigma-Aldrich/Merck (MI00300-1SET |
| MISSION LentiElite™ Whole-Genome shRNA Libraries | Vector (20,000+ human genes, 125,000+ shRNA Clones) | Packed lentiviral library (2^nd^ gen. pri-miR-based)** | Sigma-Aldrich/Merck (LIBHV1) |
| TRC Lentiviral Human Genome shRNA Library | The RNAi consortium (TRC) | lentiviral pLKO1 vector (1^st^ gen. pre-miR based)* | Dharmacon/GE Life Sciences (RHS4012) |
| TRC Lentiviral Mouse Genome shRNA Library | The RNAi consortium (TRC) | lentiviral pLKO1 vector (1^st^ gen. pre-miR based)* | Dharmacon/GE Life Sciences (RMM4013) |
| GIPZ Human Whole Genome shRNA Library | Cold Spring Harbor Labs | pGIPZ lentiviral vector, TurboGFP marks cells expressing shRNA (2^nd^ gen. pri-miR-based) ** | Dharmacon/GE Life Sciences (RHS6037) |
| GIPZ Mouse Annotated Genes shRNA Library | Cold Spring Harbor Labs | pGIPZ lentiviral vector, TurboGFP marks cells expressing shRNA (2^nd^ gen. pri-miR-based) ** | Dharmacon/GE Life Sciences (RMM5104) |
|  |  |  |  |
|  |  |  |  |

**Notes**: small interfering RNAs (siRNAs), short hairpin RNAs (shRNAs), endoribonuclease-prepared siRNAs (esiRNAs), Lentiviral (LV), Retroviral (RV), Not Available (NA).
